# Structure and Properties of Ti/Ti64 Graded Material Manufactured by Laser Powder Bed Fusion

**DOI:** 10.3390/ma14206140

**Published:** 2021-10-16

**Authors:** Evgenii Borisov, Igor Polozov, Kirill Starikov, Anatoly Popovich, Vadim Sufiiarov

**Affiliations:** Institute of Machinery, Materials, and Transport, Peter the Great St. Petersburg Polytechnic University (SPbPU), Polytechnicheskaya 29, 195251 St. Petersburg, Russia; evgenii.borisov@icloud.com (E.B.); kirill.starikov@gmail.com (K.S.); director@immet.spbstu.ru (A.P.); vadim.spbstu@yandex.ru (V.S.)

**Keywords:** additive manufacturing, selective laser melting, titanium alloys, multimaterial 3D printing, graded materials

## Abstract

Multimaterial additive manufacturing is an attractive way of producing parts with improved functional properties by combining materials with different properties within a single part. Pure Ti provides a high ductility and an improved corrosion resistance, while the Ti64 alloy has a higher strength. The combination of these alloys within a single part using additive manufacturing can be used to produce advanced multimaterial components. This work explores the multimaterial Laser Powder Bed Fusion (L-PBF) of Ti/Ti64 graded material. The microstructure and mechanical properties of Ti/Ti64-graded samples fabricated by L-PBF with different geometries of the graded zones, as well as different effects of heat treatment and hot isostatic pressing on the microstructure of the bimetallic Ti/Ti64 samples, were investigated. The transition zone microstructure has a distinct character and does not undergo significant changes during heat treatment and hot isostatic pressing. The tensile tests of Ti/Ti64 samples showed that when the Ti64 zones were located along the sample, the ratio of cross-sections has a greater influence on the mechanical properties than their shape and location. The presented results of the investigation of the graded Ti/Ti64 samples allow tailoring properties for the possible applications of multimaterial parts.

## 1. Introduction

With the advent of Additive Manufacturing (AM) technologies, it has become possible for designers to improve the technological and functional capabilities of parts by evolutionary design optimization [1,2]. AM technologies have simplified the manufacturing of complex, single-piece products, while opening up the possibility of shaping a specific, given structure [3,4,5]. One method of part optimization is the use of multiple materials in the fabrication of a single part [6,7,8]. For example, in a part that is only partially exposed to high temperatures, it is possible to use heat-resistant materials only in the temperature-loaded part. In this case, for the formation of the remaining volume of the part it is reasonable to use less heat-resistant and, at the same time, cheaper materials. In addition, the combination of strong and ductile materials is widely used, for example, in tools for machining and gears, etc. [9,10]. In implants, a very important parameter is the mechanical strength and elasticity of the material. On the one hand, it is necessary to ensure a sufficient strength to avoid fracture. On the other hand, too much elasticity of the material can lead to bone damage due to permanent differences in the strain under load [11].

Recently, an increasing number of studies have appeared in the field of forming parts from several materials during the Laser Powder Bed Fusion (L-PBF) process. The main difficulty of this process is related to the fact that the existing equipment is not designed to use more than one powder material simultaneously. Therefore, much research on the development and modification of the equipment is being carried out [12,13,14,15,16]. For the developers, the main difficulty to overcome is the necessity to apply a thin layer of powder material of the heterogeneous chemical composition. At the same time, this heterogeneity must correspond to the computer model of the part on each layer.

Another important direction of research is the study of microstructures and the properties of the multi-material products themselves, which are obtained using the L-PBF [17,18,19,20]. In these works, the microstructure and continuity of the transition zone, its phase composition, and mechanical characteristics were investigated.

Currently, there are several research papers devoted to the L-PBF of parts with a graded composition by changing the feedstock powder to build separate parts of a specimen. For example, alternatively using powders of different compositions was applied to fabricate CuSn/18Ni300 [18], NiTi/Ti6Al4V [19], AlSi10Mg/Cr18Ni10Ti stainless steel [21], 316L/CuSn10 [22], or 316L/Cu [23] graded specimens by L-PBF. The authors of [20] investigated the possibility of using the L-PBF process to fabricate graded samples using In718 and Ti6Al4V powders utilizing intermediate layers of mixed powders with a different ratio. The first commercial multimaterial recoating system for the L-PBF machines was recently introduced by Aerosint [24,25] suggesting the importance of multimaterial AM development. It uses mechanical forces to hold the powder on the drum and can release it at the desired location, generating a 2D single material image in a line-by-line manner. Currently, its possibilities have been demonstrated by 3D printing a copper alloy/steel bi-metallic parts [26].

For medical applications, Ti and Ti64 alloys have their advantages and disadvantages. The Ti64 alloy has great strength, but pure Ti has a great resistance to corrosion [27] and does not contain toxic impurities (Vanadium). Therefore, the formation of a graded part, where the advantages of both alloys are used, is relevant.

This work aimed to investigate the microstructure and mechanical properties of Ti/Ti64 graded samples fabricated by L-PBF with different geometries of the graded zones, as well as effects of heat treatment and hot isostatic pressing on the microstructure of the bimetallic Ti/Ti64 samples.

## 2. Materials and Methods

**Figure 1:**
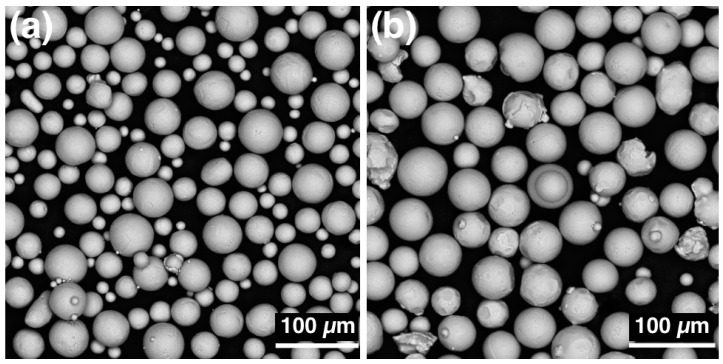
Scanning electron microscope (SEM)-images of (**a**) Ti and (**b**) Ti64 powders.

Commercially available CP-Ti (grade 2) and Ti-6Al-4V (Ti64, grade 5) alloy powders (Normin LLC, Borovichi, Russia) obtained by plasma atomization process were used as the feedstock material to fabricate the samples. The particles of both powders were spherical shaped (Figure 1) and had a mean size of d_50_ = 34 µm and d_50_ = 47 µm for Ti and Ti64 alloys, respectively.

The samples were fabricated using the SLM Solutions 280HL machine (Lübeck, Germany) in an argon atmosphere (99.99% purity) on a Ti64 built substrate. For microstructural characterization and microhardness evaluation, the samples of 20 mm height and 15 × 15 mm^2^ section were built. Initially, one of the feedstock materials was used during the L-PBF process to fabricate the first half of the sample (10 mm). After that, the powder in the machine was changed to the second material and the second half of the sample was manufactured. The samples for mechanical tests were fabricated using a similar technique by changing the powder in the machine after building part of the sample. The same L-PBF process parameters were used for Ti and Ti64 alloys that were chosen based on the previous studies [28,29]. The following process parameters were used: scanning speed—805 mm/s, laser power—275 W, hatch distance—120 µm, layer thickness—50 µm. The laser beam size was approximately 80 µm.

Heat treatment of the samples was carried out using a vacuum furnace (Carbolite Gero GmbH & Co. KG, Neuhausen, Germany) at 10^-3^–10^-4^ mbar at 950 °C for 2 h, followed by furnace cooling. The regime was chosen based on AMS-H-81200A specification for the Ti64 alloy. The same temperature was used for hot isostatic pressing (HIP) of the graded samples, while the pressure was 100 MPa.

The microstructure was studied using a Leica DMI 5000 (Leica Microsystems, Wetzlar, Germany) optical microscope. To study the chemical composition, a Mira 3 (TESCAN, Brno, Czech Republic) scanning electron microscope with an energy dispersive X-ray (EDX) spectroscopy module was used.

Ti/Ti64 samples were scanned on a v|tome|x m300 X-ray computer tomography (CT). The system was equipped with an X-ray source with a maximum voltage of 300 kV. The obtained data were processed and visualized using an extended software package AVIZO for three-dimensional analysis and voxels visualization. Segmentation was performed using global and local gray thresholds.

The hardness of the samples was measured using Zwick/Roell ZHU 250 tester (Zwick GmbH, Ulm, Germany) with a Vickers intender along the material transition area.

**Figure 2:**
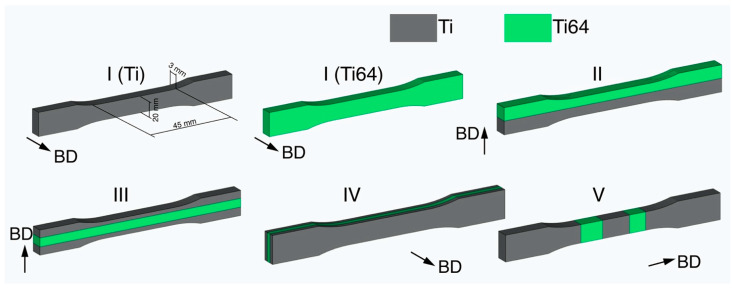
Schematic representation of tensile specimens’ configuration.

Tensile tests were carried out at room temperature using Zwick/Roell Z050 (Zwick GmbH, Ulm, Germany) testing machine. Figure 2 schematically shows the samples used for tensile tests of the graded materials. The gauge length was 45 mm and the width was 20 mm, while the thickness of the specimens was 3 mm. Three samples per point were used for the tensile tests.

Specimens consisting entirely of Ti of Ti64 materials were labeled as I (Ti) and I (Ti64), respectively. The remaining specimens consisting of two materials are shown in Figure 2. Type II specimens were split in half and consisted of 50% Ti and 50% Ti64 materials. Type III and IV had an insert from Ti64 alloy located at the center of the specimen with different orientations of the insert with 50%/50% volume fraction of the alloys. The IV type specimen had two inserts from Ti64 alloy as shown in Figure 2.

## 3. Results and Discussion

**Figure 3:**
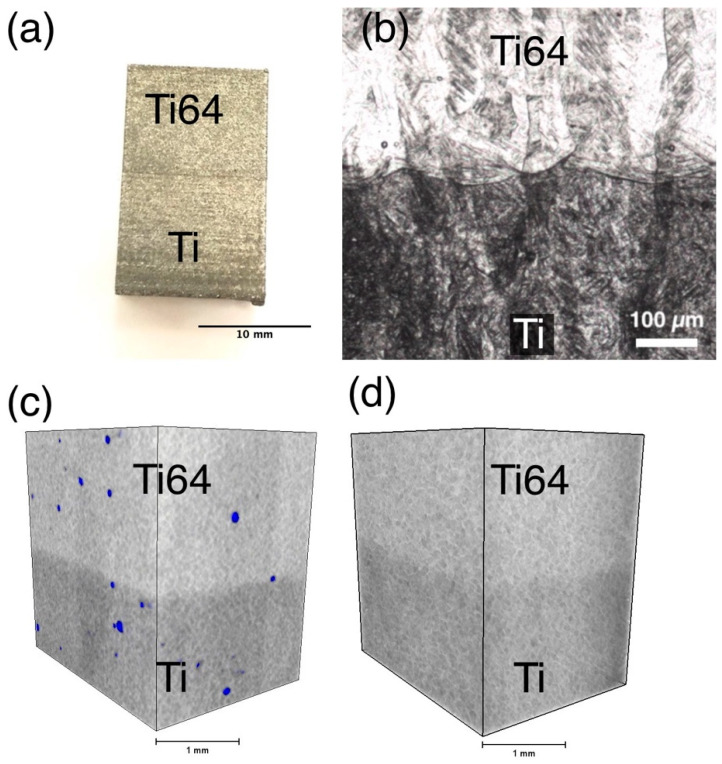
(**a**) A photograph of the Ti/Ti64 sample, (**b**) microstructure of the transition zone, (**c**) as-built, and (**d**) HIPed specimens’ volume obtained by CT-reconstruction.

A sample consisting of Ti and Ti64 materials fabricated by L-PBF is shown in Figure 3a. There are no visible differences between the zones of the sample externally. They have the same color and surface roughness. A small line along the alloy interface, caused by the thermal expansion of the lower zone during fabrication, is noticeable. An image of the transition zone section of the sample in the initial state obtained by computed tomography (Figure 3c) shows internal defects in the form of pores, the average size of which is about 50 µm. It is also possible to see the transition from one material to another using the computed tomography, as the Ti64 material has a lighter shade compared to pure Ti due to the difference in density. The residual pores in the material after the HIP were not detected by CT, but the transition zone from one material to the other can also be seen (Figure 3d).

The microstructure of the transition zone between the two materials is shown in Figure 3b. No visible defects in the form of a lack of fusion or cracks were found. After etching, a distinct transition zone can be seen between Ti and Ti64 zones. It can be seen that the transition zone has a thickness of about 50–100 µm, which corresponds to the 1–2 layer thickness used during the L-PBF process.

The microstructure of the Ti zone consists of fine martensitic α’ needles, while the Ti64 zone exhibits a needle-like martensitic α’ phase within the columnar primary β grains. The high solidification rates typical for the L-PBF process resulted in a metastable microstructure in the case of both alloys. Titanium undergoes an α → β phase transformation above 890 °C, and this allotropic phase transformation affects the microstructure and texture of the material. The Ti64 alloy undergoes an β ↔ α + β phase transformation at about 1000 °C [30]. However, the L-PBF process leads to metastable martensitic microstructure due to the high cooling rates up to 10^5^ K/s [31]. The L-PBF process, accompanied by rapid solidification, leads to the formation of a martensitic microstructure with elongated grains of the primary β-phase filled with the finely dispersed lamellar α-phase. The partial remelting of the previous layers provides the epitaxial growth of such grains.

**Figure 4:**
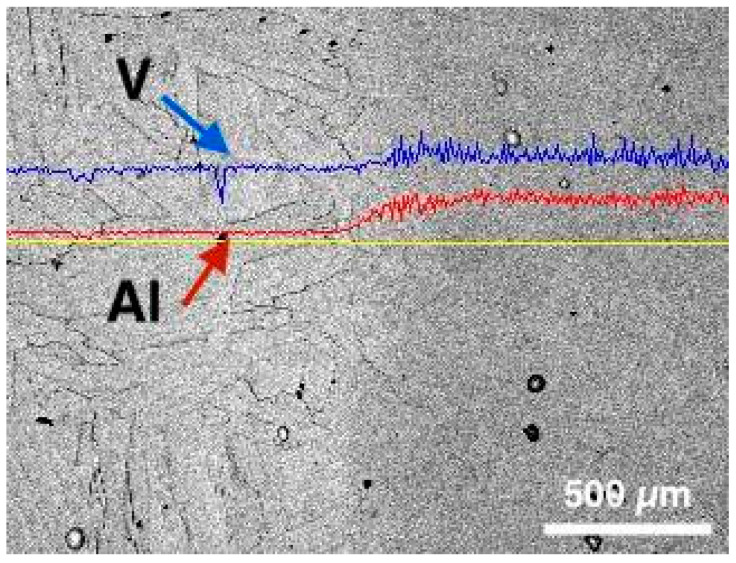
EDX results showing the change of V and Al composition distribution along the transition zone from Ti (**left**) to Ti64 (**right**) on the sample.

The results of a study of the chemical composition of the transition zone (Figure 4) showed that the Al and V content increases smoothly from the zone of Ti to the zone of Ti64. According to the measurement results, the width of the transition zone can be estimated at approximately 200 μm.

Depending on the heat treatment temperature and cooling rate, the titanium microstructure may have different morphology: equiaxed α-Ti grains inside the primary β-grains, a Widmanstett structure, and a lamellar or needle morphology of the α-phase [32].

**Figure 5:**
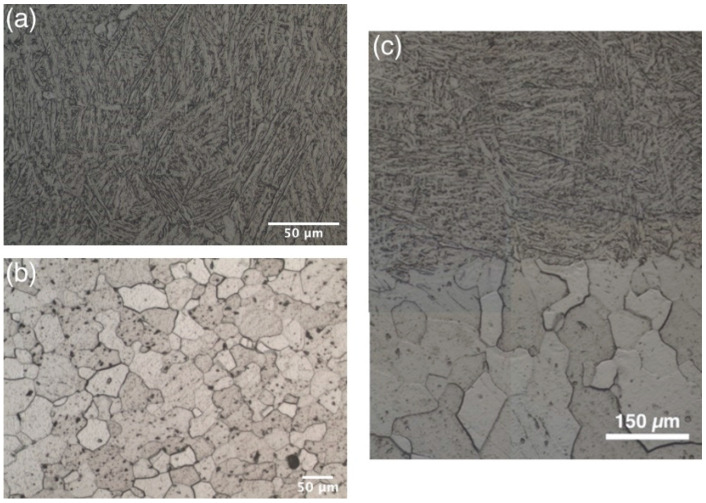
Microstructures of Ti/Ti64 sample after heat treatment: (**a**) Ti64 zone, (**b**) Ti zone, (**c**) the transition zone.

The heat treatment of the Ti/Ti64 sample at 950 °C resulted in the transition of the martensitic α’-phase to α + β phase in the case of Ti64 zone and α-Ti phase grains in the case of Ti zone (Figure 5).

After heat treatment, the Ti zone consists of equiaxed α-Ti grains, which indicate the recrystallization processes [33]. The recrystallization process led to the disappearance of the preferential orientation of the grains. They had an equiaxial shape and size from 80 to 150 µm.

**Figure 6:**
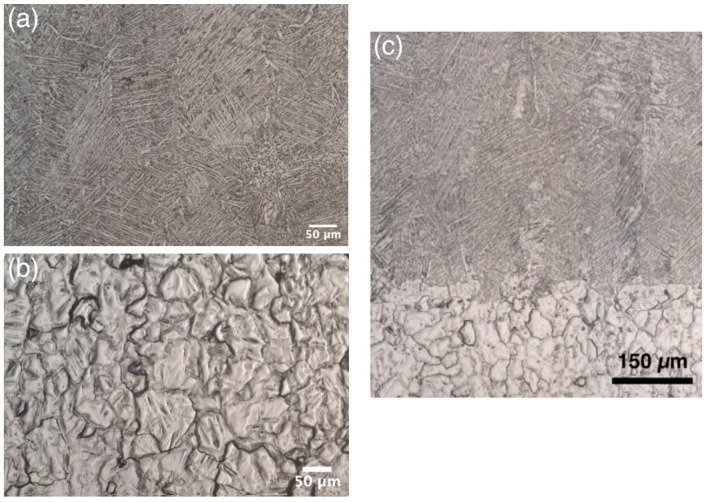
Microstructures of Ti/Ti64 sample after HIP: (**a**) Ti64 zone, (**b**) Ti zone, and (**c**) the transition zone.

After HIP, the microstructure of the samples underwent changes similar to those after the heat treatment, but there were differences in the morphology. The microstructure of the Ti64 alloy zone (Figure 6a) also consists of α + β phases with lamellar morphology, formed as a result of martensitic α’-phase decomposition to α + β. The formation of the lamellar α-phase with a larger lamellar size and β-phase grains occurs in the Ti64 alloy, in comparison to heat-treated conditions. The increase in the α-phase lamellar size occurs both within and along the grain boundaries, which may lead to an increase in ductility as deformation is mainly found along the grain boundaries.

The Ti zone (Figure 6b) after HIP has a microstructure of equiaxed α-Ti grains with larger sizes compared to heat-treated ones, which may be caused by the differences in the cooling rate at a different post-processing [29].

**Figure 7:**
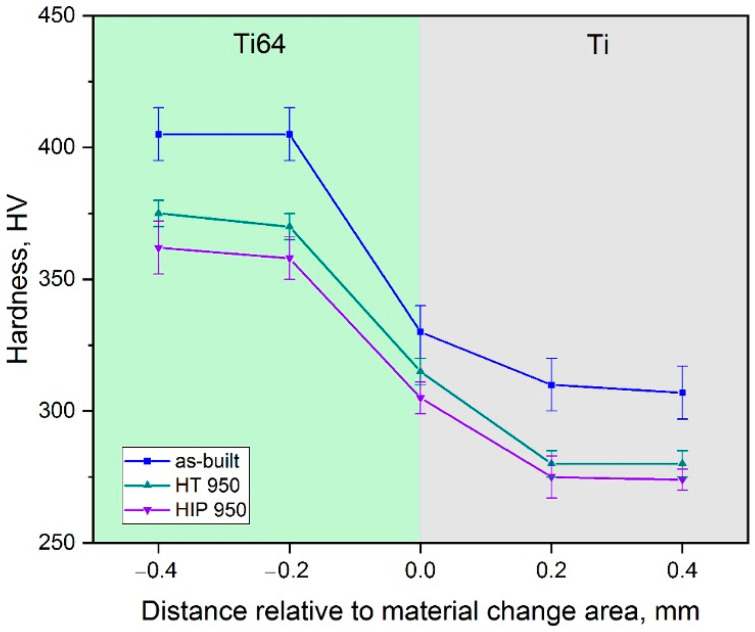
Hardness distribution for Ti/Ti64 samples along the material change area for as-built, heat treated, and HIPed conditions.

Figure 7 shows hardness distribution for the Ti/Ti64 samples along the material transition area of as-built, heat-treated, and HIPed samples.

The hardness of the Ti64 zone is higher compared to the Ti zone for all tested conditions. The as-built condition exhibited the highest hardness values for both the Ti and Ti64 zone due to metastable microstructures formed during the L-PBF process. After heat treatment and hot isostatic pressing, the hardness values decreased for both the Ti and Ti64 zones due to the stress relieving and martensite phase decomposition. Due to different cooling conditions in the vacuum furnace and with HIP, there were differences between the values of hardness. After HIP, the microstructure was slightly coarser in terms of grain and lamellar size compared to the heat-treated samples, which resulted in lower hardness values along all the zones for the HIPed sample.

Tensile tests of pure Ti, Ti64, and graded Ti/Ti64 material have been made for samples fabricated by L-PBF and subsequent hot isostatic pressing. The results are summarized in Table 1.

The values of yield and tensile strengths of all the graded samples were between the values for pure Ti and Ti64. The samples of the V type had the lowest strength values; this type had 4 Ti/Ti64 material change interfaces which transversely directed the forces applied during the tensile test. The type II samples turned out to be more strengthened; this type had one interface of Ti/Ti64 material changing, directed along the axis of the tensile during the test. Even the higher strength values were demonstrated by type III samples. This type was characterized by the 2 Ti/Ti64 material change interfaces along the axis of the tensile and the total area of the interfaces were twice as large as those of type II. The highest strength characteristics between the investigated graded materials were found in the type IV samples. The geometry of this sample type had 2 Ti/Ti64 material change interfaces along the axis of the tensile with the largest contact area between the different materials.

The graded samples did not exhibit high elongation values. The changes in the elongation values for different types of samples have a similar tendency to the strength values. The fracture of the type V samples occurred at the material interface. This was potentially due to a partial oxidation of the metal surface, or the cooling of the specimens during a material change. Other sample types had interfaces along the axis of the tensile, as well as a low elongation. Therefore, another possible reason for the low elongation may be the Ti64 zones having a higher yield strength which could limit the elongation of the Ti zones and lead to the formation and failure of stress concentrators with relatively low values of elongation.

It should be noted that the homogeneous specimens, as well as the type V specimens, were fabricated with a build direction along the tensile load, and the other specimen types were fabricated with a build direction perpendicular to the tensile load. As shown in the previous research [34], the strength of the horizontally fabricated samples was higher compared to the vertically built samples, which could contribute to the lower properties of the type V specimens.

The presented results of the investigation of the graded Ti/Ti64 samples allowed tailoring properties for possible applications of multimaterial parts.

## 4. Conclusions

The investigation of samples with the graded chemical composition, Ti/Ti64, was presented in this work. The study of the transition zone structure showed that these samples had a distinct character and did not undergo significant changes during heat treatment and hot isostatic pressing. 

The study of tensile mechanical properties showed that, when the zones are located along the sample, the ratio of cross-sections has a greater influence on the mechanical properties than their shape and location. When the zones are arranged transversely to the specimen, a failure occurs at the interface and the relative elongation is extremely low. Future investigations in multimaterial 3D printing must pay attention to the possibility of creating change interfaces with the smooth changing of chemical compositions and an increasing transient zone.

## Figures and Tables

**Table 1:** The results of tensile tests of Ti, Ti64, and graded Ti/Ti64 samples produced by L-PBF with the subsequent HIP.

Specimen Type	Tensile Strength, MPa	Yield Strength, MPa	Elongation at Break, %
I (Ti)	700 ± 12	596 ± 8	16 ± 4
I (Ti64)	998 ± 21	821 ± 11	10 ± 3
II	728 ± 29	693 ± 25	3 ± 1
III	760 ± 14	726 ± 11	5 ± 2
IV	839 ± 10	754 ± 8	7 ± 3
V	703 ± 36	667 ± 32	2 ± 1

## Data Availability

The data presented in this study are available on request from the corresponding author.
